# Nature of Long-Lived Moiré Interlayer Excitons in Electrically Tunable MoS_2_/MoSe_2_ Heterobilayers

**DOI:** 10.1021/acs.nanolett.4c02635

**Published:** 2024-08-30

**Authors:** Evgeny M. Alexeev, Carola M. Purser, Carmem M. Gilardoni, James Kerfoot, Hao Chen, Alisson R. Cadore, Bárbara L.T. Rosa, Matthew S. G. Feuer, Evans Javary, Patrick Hays, Kenji Watanabe, Takashi Taniguchi, Seth Ariel Tongay, Dhiren M. Kara, Mete Atatüre, Andrea C. Ferrari

**Affiliations:** † Cambridge Graphene Centre, University of Cambridge, 9 JJ Thomson Avenue, CB3 0FA Cambridge, U.K.; ‡ Cavendish Laboratory, University of Cambridge, JJ Thomson Avenue, Cambridge CB3 0HE, U.K.; ¶ Brazilian Nanotechnology National Laboratory (LNNano), Brazilian Center for Research in Energy and Materials (CNPEM), Campinas, 13083-849 Sao Paulo, Brazil; § École Normale Supérieure, PSL, 5 Rue D’ulm, Paris 75005, France; ∥ Materials Science and Engineering, School for Engineering of Matter,Transport and Energy, Arizona State University, Tempe, Arizona 85287, United States; ⊥ Research Center for Electronic and Optical Materials, National Institute for Materials Science, 1-1 Namiki, Tsukuba 305-0044, Japan; # Research Center for Materials Nanoarchitectonics, National Institute for Materials Science, 1-1 Namiki, Tsukuba 305-0044, Japan

**Keywords:** layered materials heterostructures, transition-metal dichalcogenides, interlayer excitons, moiré superlattice, valley polarization, Stark shift, photoluminescence

## Abstract

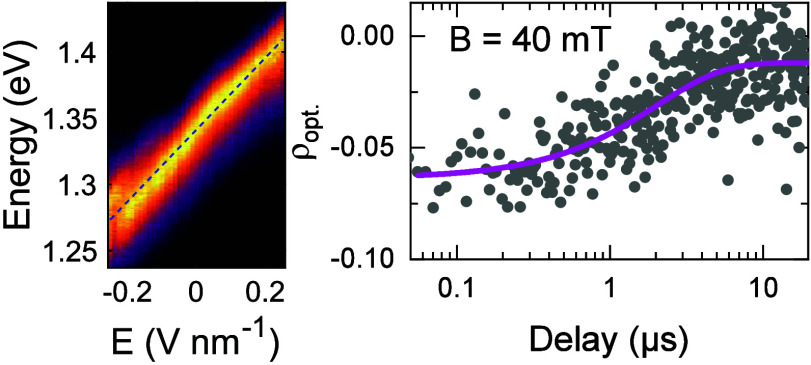

Interlayer excitons in transition-metal dichalcogenide heterobilayers combine high binding energy and valley-contrasting physics with a long optical lifetime and strong dipolar character. Their permanent electric dipole enables electric-field control of the emission energy, lifetime, and location. Device material and geometry impact the nature of the interlayer excitons via their real- and momentum-space configurations. Here, we show that interlayer excitons in MoS_2_/MoSe_2_ heterobilayers are formed by charge carriers residing at the Brillouin zone edges, with negligible interlayer hybridization. We find that the moiré superlattice leads to the reversal of the valley-dependent optical selection rules, yielding a positively valued g-factor and cross-polarized photoluminescence. Time-resolved photoluminescence measurements reveal that the interlayer exciton population retains the optically induced valley polarization throughout its microsecond-long lifetime. The combination of a long optical lifetime and valley polarization retention makes MoS_2_/MoSe_2_ heterobilayers a promising platform for studying fundamental bosonic interactions and developing excitonic circuits for optical information processing.

Layered materials heterostructures (LMHs) comprising monolayer transition-metal dichalcogenides (1L-TMDs) are promising platforms for optoelectronics^1−4^ and quantum technology^5^ as they combine optically addressable spin and valley degrees of freedom^6−8^ with unique tunability through the choice of material combination^9−11^ and rotational alignment.^12−14^ TMD heterobilayers have drawn particular interest due to their ability to host interlayer excitons (iXs)^15,16^ which offer lifetime approaching 200 μs,^17^ strong repulsive dipolar interaction,^18,19^ and high sensitivity to rotational alignment,^20−22^ strain,^17,23^ and electric^24^ and magnetic^25^ fields. Different TMD combinations give rise to iX with drastically different properties, including oscillator strength,^26,27^ center-of-mass momentum,^20,21^ and degree of interlayer hybridization.^19,22^ Of the plethora of possible TMD combinations, the majority of research effort focused on 1L-MoSe_2_/1L-WSe_2_^27−30^ and 1L-WS_2_/1L-WSe_2_.^31−33^ For other material combinations, key aspects of the iX nature, such as real- and momentum-space configuration, remain elusive due to the complexity of the underlying physics.

In this work, we investigate iX in 1L-MoS_2_/1L-MoSe_2_ using polarization-resolved magneto-photoluminescence spectroscopy. We find that iX photoluminescence (PL) is visible only in devices with relative twist angle less than 5°. This indicates that the constituent iX charge carriers reside at the edges of the Brillouin zone. We study the iX PL response to out-of-plane electric and magnetic fields and show that iX is formed by charge carriers at the ±K valleys with negligible degree of interlayer hybridization. Our time- and polarization-resolved PL measurements reveal microsecond-scale retention of optically induced valley polarization, demonstrating the potential of 1L-MoS_2_/1L-MoSe_2_ for opto-valleytronic applications.

Figure 1a shows an optical microscope image of one of our electric-field-tunable 1L-MoS_2_/1L-MoSe_2_. The hexagonal boron nitride (hBN) layers provide a flat and clean dielectric environment for 1L-MoS_2_/1L-MoSe_2_, and the transparent few-layer graphene (FLG) top and bottom gates allow optical measurements under an out-of-plane electric field. Each of the eight devices is fabricated using deterministic mechanical transfer,^34,35^ with constituent monolayers obtained through micromechanical exfoliation of bulk TMD crystals prepared by flux zone growth. Thickness and quality of constituent layers are characterized using Raman^36^ and PL spectroscopy (see Methods and Supporting Information Figures S1, S2). Figure 1b presents a schematic of the type-II alignment of electronic bands within 1L-MoS_2_/1L-MoSe_2_, with conduction-band minimum (valence-band maximum) occurring in 1L-MoS_2_ (1L-MoSe_2_).^37,38^ The type-II band alignment leads to interlayer charge separation and the formation of iX, with PL lower in energy compared to the intralayer PL of the constituent monolayers. The devices offer a range of twist angles between the 1L-TMD θ, enabling the investigation of iX momentum-space configuration. Figure 1c compares room-temperature (RT) PL spectra of two devices with θ = 1° (top) and θ = 28° (bottom). We identify θ using polarization-resolved second-harmonic generation (SHG) (Figure 1d) and note that our measurements do not allow us to distinguish parallel from antiparallel alignment between monolayers (Supporting Information Figure S3). Both devices show PL peaks corresponding to the A exciton in 1L-MoSe_2_ (1L-MoSe_2_ X_A_) at 1.55 eV and the A and B excitons in 1L-MoS_2_ (1L-MoS_2_ X_A_ and X_B_) at 1.85 and 2.0 eV, respectively.^39,40^ Crucially, the iX PL peak at ∼1.3 eV is visible only in the device with θ = 1°. Of the eight devices with θ ranging from 1° to 28°, only those with θ ≤ 5° reveal the iX PL peak at RT (Supporting Information Figure S4), consistent with ref (41). Thus, close rotational alignment is critical for the observation of iX in 1L-MoS_2_/1L-MoSe_2_.

**Figure 1:**
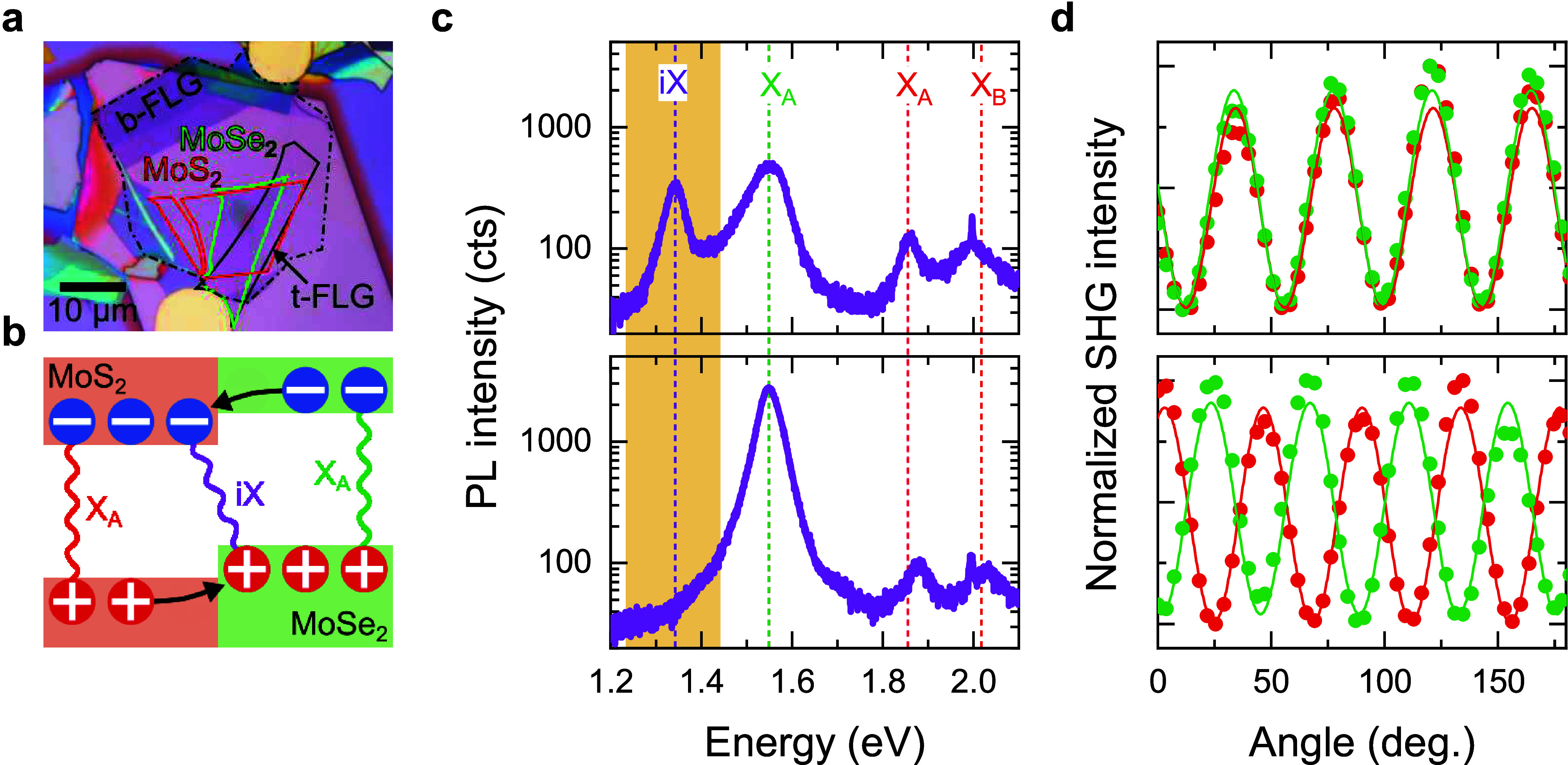
**iX in 1L-MoS**_**2**_**/1L-MoSe**_**2**_. (a) Optical microscope image of an electrically tunable 1L-MoS_2_/1L-MoSe_2_ device. 1L-MoS_2_ and 1L-MoSe_2_ regions are outlined in red and green, respectively. Solid (dashed) black lines show the position of top (bottom) FLG gates. (b) Schematic band alignment of 1L-MoS_2_ and 1L-MoSe_2_. (c) RT PL spectra recorded in two devices with different θ. The closely rotationally aligned device (top panel, θ = 1°) shows intralayer 1L-MoS_2_ B and A excitons and 1L-MoSe_2_ A exciton peaks, as well as an iX peak appearing in a lower-energy range (highlighted in copper), not visible in the PL spectrum of the strongly misaligned device (bottom panel, θ = 28°). (d) Polarization-resolved SHG intensity recorded in isolated (red) 1L-MoS_2_ and (green) 1L-MoSe_2_ regions of the two devices, confirming θ = 1° (top) and θ = 28° (bottom).

The ∼3.7% mismatch in lattice constants of 1L-MoS_2_ and 1L-MoSe_2_^42^ eliminates θ dependence of the interlayer distance as an underlying source of this behavior.^43^ Instead, the high sensitivity of the iX PL intensity to θ indicates that iX is formed by the charge carriers residing in valleys at the edges of the Brillouin zone (BZ). Homo- and heterobilayers where at least one of the charge carriers resides at the Γ valley at the BZ center of display iX PL throughout the entire θ range, as the momentum-space separation between electron and hole remains unchanged.^21,44,45^ In contrast, in heterobilayers where both constituent charges reside at the BZ edges, large momentum-space separation of electron and hole suppresses radiative recombination of iX in devices with θ away from 0 or 60°,^13,20^ consistent with our observations.

Our device structure enables control of doping and the electric field independently. We use this to identify the real-space configuration of iX by studying its response to an out-of-plane electric field in the neutral regime. We note that the doping dependence of iX emission is reminiscent of what is observed in 1L-WS_2_/1L-WSe_2_^19,46,47^ (Supporting Information Figure S5). Figure 2a presents the normalized iX PL spectrum recorded as a function of electric field at 4K. The iX PL energy shifts linearly with a rate ∼0.31 eV nm V^–1^ and can be tuned over a 144-meV range within the gate tuning limits of our device. We find an average tuning response across three devices of ∼0.30 eV nm V^–1^, yielding an average dipole size ∼0.55(3) nm^19,24^ (Supporting Information Figure S6), in good agreement with the ∼0.6 nm separation between the layers.^48^ A similar dipole size was observed in 1L-MoSe_2_/1L-WSe_2_^19^, where iX is formed by nonhybridized electrons and holes, while MoSe_2_ homobilayers show a reduced dipole size of 0.26 nm due to charge-carrier hybridization.^49^ Comparatively, our results suggest negligible interlayer hybridization for our devices.

**Figure 2:**
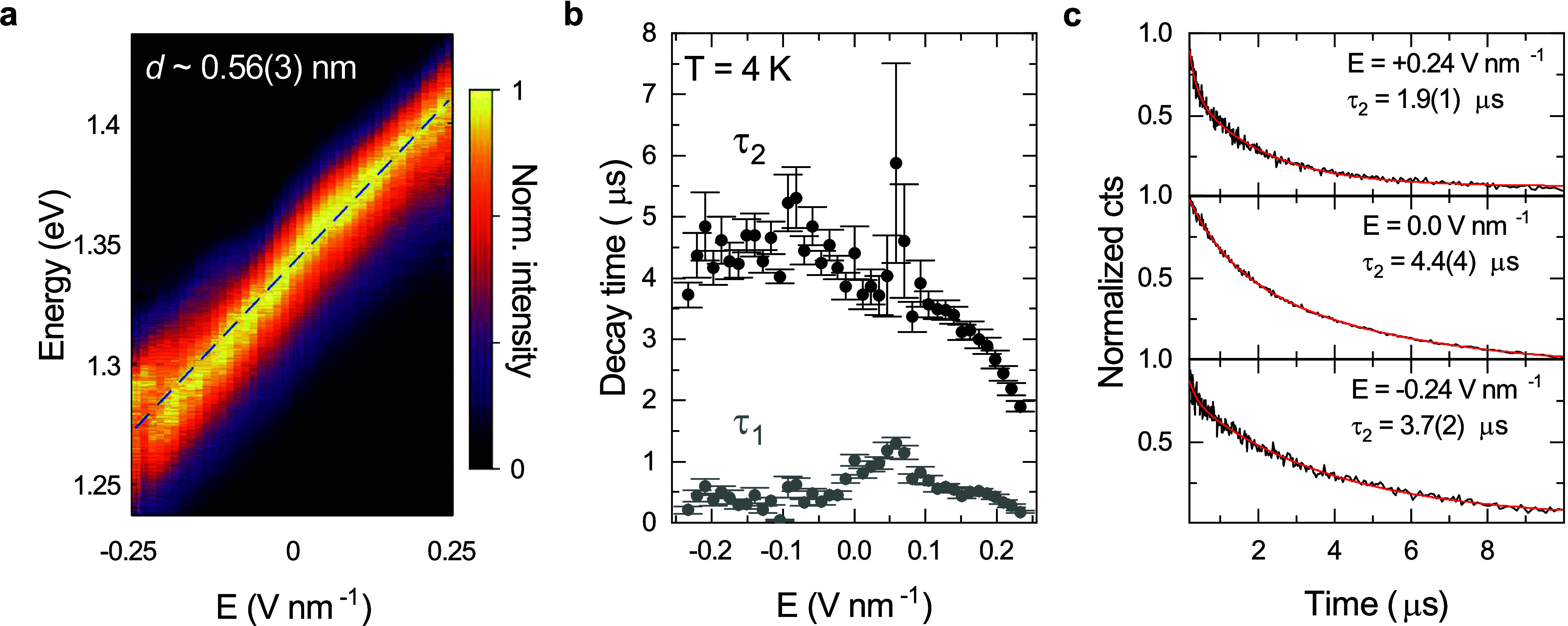
**Electric field tuning of iX.** (a) Normalized iX PL under out-of-plane electric field. The iX PL energy shows a linear shift with slope ∼0.31 eV nm V^–1^, corresponding to a dipole size of *d* = 0.56(3) nm, closely matching the expected interlayer distance. (b) Variation of iX PL decay time as a function of electric field. Gray and black circles correspond to fast (τ_1_) and slow (τ_2_) time constants, respectively. (c) PL decay acquired at +0.24, 0, and −0.24 V nm^–1^. Red curves are biexponential fits to the data.

Figure 2b shows the iX PL decay time constants as a function of electric field. We extract these constants from a biexponential fit to the time-resolved PL. Figure 2c presents examples of a PL decay trace recorded at three applied field values along with their corresponding fit curves. We observe a microsecond-long iX lifetime, with a fast time constant τ_1_ = 1.0(1) μs and a slow time constant τ_2_ = 4.4(4) μs at zero electric field—an order of magnitude longer than typical lifetimes of 10–100 ns reported for 1L-MoSe_2_/1L-WSe_2_.^13^ The slow time constant in other devices ranges from 0.1 to 3.0 μs (Supporting Information Figure S7). The variability of PL lifetime measured across different devices supports the assignment of this time scale to iX PL, rather than defect-bound PL. The fast time constant is mostly field-independent, except for ∼0.06 V nm^–1^, where it increases to 1.3 μs. Shortening of τ_1_ for the electric field away from this value is likely caused by inadvertent electrostatic doping induced by a slight asymmetry in the thicknesses of the bottom and top dielectric layers. The slow time constant τ_2_ shows a gradual decrease with increasing electric field, consistent with a change in radiative lifetime due to a field-induced variation of electron–hole separation. For electric field antiparallel to the iX electric dipole moment, the separation between the two charge carriers is reduced, leading to an increased probability of radiative recombination. The opposite process takes place for parallel field alignment. That said, the PL decay time remains slow (τ_1_ ≥ 0.04 μs, τ_2_ ≥ 1.9 μs) throughout the entire field-tuning range.

We use polarization-resolved magneto-PL spectroscopy to identify the valley configuration of iX. Figure 3a shows iX PL spectra recorded using right circularly polarized (σ^+^), 1.94 eV excitation as a function of applied out-of-plane magnetic field *B* ranging from −6 to 6 T; blue (red) curves correspond to PL with σ^+^ (σ^–^) polarization. The iX PL remains cross-polarized with respect to the excitation laser throughout the entire magnetic field range. Two distinct mechanisms are known to give rise to this behavior in TMD heterobilayers: 1) directional intervalley scattering^50^; 2) moiré-induced reversal of the valley-dependent optical selection rules.^51^

**Figure 3:**
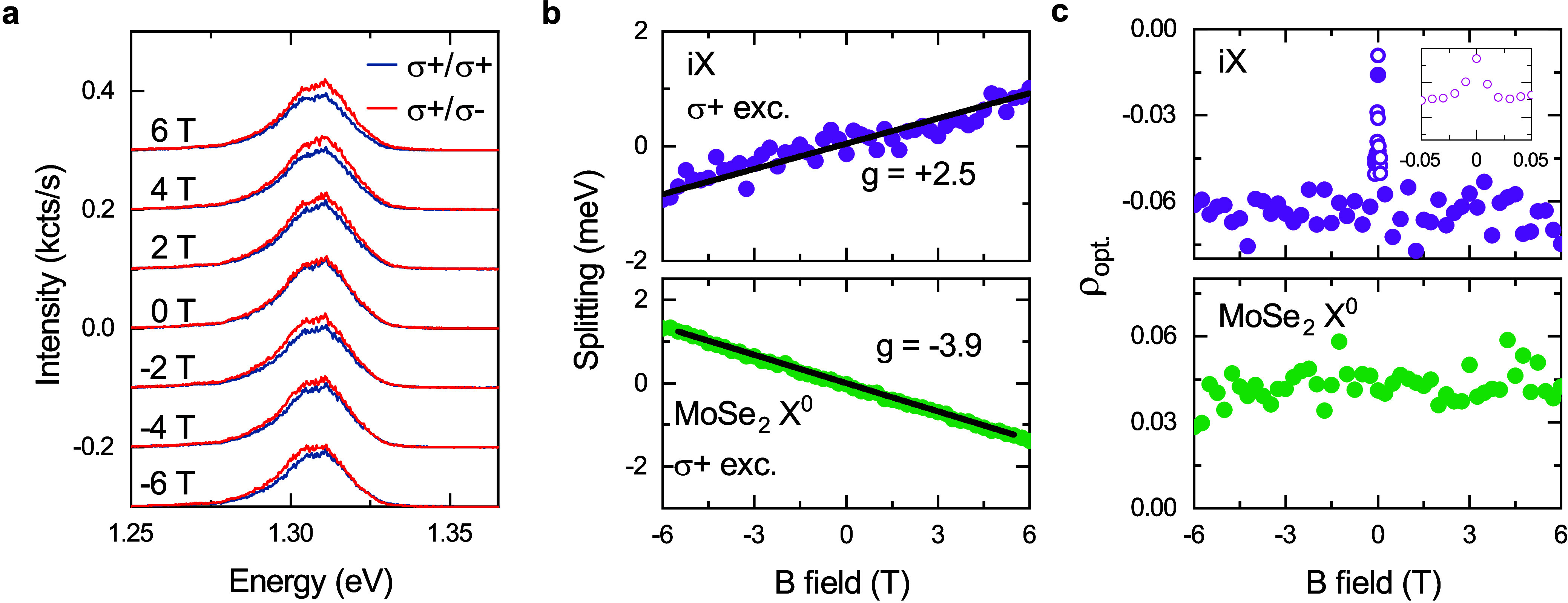
**Magneto-PL spectroscopy of iX.** (a) Helicity-resolved iX PL spectra recorded under out-of-plane magnetic field ranging from −6 to 6 T using σ^+^ polarized 1.94 eV optical excitation; blue (red) curves correspond to PL with σ^+^ (σ^–^) polarization. (b) Energy splitting between σ^+^ and σ^–^ polarized PL as a function of out-of-plane magnetic field for (top) iX in heterobilayer and (bottom) neutral excitons (X^0^) in 1L-MoSe_2_ regions. Landé g-factors extracted using linear fits are listed next to each plot. (c) Optically induced valley polarization calculated as ρ_opt_ = (*I*^++^ + *I*^––^ – *I*^+–^ – *I*^–+^)/(*I*^++^ + *I*^––^ + *I*^+–^ + *I*^–+^) as a function of magnetic field for iX (top) and 1L-MoSe_2_ X^0^ (bottom), where *I*^*XY*^ is the intensity of PL with σ^*Y*^-polarization collected under σ^*X*^-polarized excitation. Unfilled circles in the panel and the inset are extracted from a fine scan around 0 T, showing the small-field dependence of ρ_opt_ for iX.

We identify the underlying mechanism in 1LMoS_2_/1L-MoSe_2_ based on the sign of the energy splitting between σ^+^ and σ^–^ polarized PL under magnetic field. Figure 3b is a plot of the energy splitting (Δ*E*) as a function of *B* for the iX in the heterobilayer region (top panel) and the neutral intralayer excitons (X^0^) in an isolated 1L-MoSe_2_ region (bottom panel). We define Δ*E* as *E*_σ^+^_ – *E*_σ^–^_ = *gμ*_B_*B*, where *E*_σ^+^_(*E*_σ^–^_) is the energy of the σ^+^ (σ^–^) polarized PL, *g* is the effective Landé g-factor, and μ_B_ is the Bohr magneton. For X^0^ in 1L-MoSe_2_, we extract *g* = −3.90(2), consistent with previous reports,^52^ where the minus sign stems from valley-Zeeman interaction and valley-dependent optical selection rules for 1L-TMDs:^53^ σ^+^-polarized light couples to optical transitions in the +K valley, which has lower energy at positive *B*. In contrast, we obtain *g* = +2.50(7) for iX—the positive sign of *g* shows that iX PL from the +K valley appears with σ^–^ polarization, confirming the reversal of optical selection rules with respect to the monolayer case. All devices show positive iX g-factors with an average value of +4.5. In TMD heterobilayers, the reversal of the selection rules arises from local changes in crystal symmetry induced by the moiré superlattice.^51^ We observe iX g-factors that are always positive, but range from +1.0 to +8.0 across devices, with variability between different positions within individual devices (see Supporting Information Figure S8). The variability is likely a consequence of the difference in the moiré superlattice parameters arising from different θ and local strain in different devices.

Two mechanisms can give rise to the observed PL polarization under finite magnetic field: 1) optically induced valley polarization^54^; 2) Zeeman-shift-induced valley thermalization.^39^ The former is limited by nondirectional intervalley scattering, while the latter arises from exciton relaxation into the lower-energy valley. We calculate the degree of optically induced valley polarization independently as , where *I*^*XY*^ represents the intensity of PL with σ^*Y*^-polarization collected under σ^*X*^-polarized excitation. Figure 3c displays the dependence of ρ_opt_ on magnetic field for iX and 1L-MoSe_2_ X^0^. 1L-MoSe_2_ X^0^ shows a constant PL polarization degree ∼4%, consistent with earlier reports.^55,56^ In contrast, |ρ_opt_| for iX shows a distinct increase with increasing |*B*|, saturating at ∼6% above ±20 mT (see inset in Figure 3c). This dependence is consistent across all devices, with |ρ_opt_| ranging from 6% to 14%. In the absence of a magnetic field, |ρ_opt_| ranges from 0% to 7%. Similar sharp changes in valley polarization degree with magnetic field have been observed for iX in 1L-MoSe_2_/1L-WSe_2_^57^ and 1L-MoS_2_/1L-WSe_2_^58^, as well as intralayer excitons in 1L-WS_2_ and 1L-WSe_2_.^59^ This effect was attributed to the suppression of intervalley scattering of intralayer excitons within the monolayer with dark excitonic ground state.^6^ However, we observe device-specific saturation field for ρ_opt_ (*B*_sat_) ranging from 0.02 to 3 T (Supporting Information Figure S9), indicating that it is not defined by the properties of individual monolayers, but the collective property of the assembled LMH.

Figure 4a presents the polarization-resolved decay of iX PL recorded at 0 and 40 mT in a device with *B*_sat_ ∼ 200 mT. The difference in intensity for cross-co polarization allows us to monitor |ρ_opt_| as a function of time. Figure 4b plots the time-resolved ρ_opt_ extracted from the iX PL decay measured at 0 and 40 mT; the solid curves are guides to the eye. Without magnetic field (black filled circles), iX has a low polarization degree (|ρ_opt_| < 2%) throughout the measurement range. In contrast, at *B* = 40 mT (gray filled circles) |ρ_opt_| starts at 6% and shows a gradual decay toward zero, with a characteristic 1/e time ∼2 μs. These results indicate that the loss of valley polarization for iX is governed by at least two processes occurring at different time scales: 1) fast intervalley relaxation with a characteristic time shorter than 10 ns (i.e., timing resolution of our measurement) dominates at zero magnetic field. 2) This process is suppressed at 40 mT, revealing a slower microsecond-scale relaxation. We note that this time scale directly reflects the loss of valley polarization.

**Figure 4:**
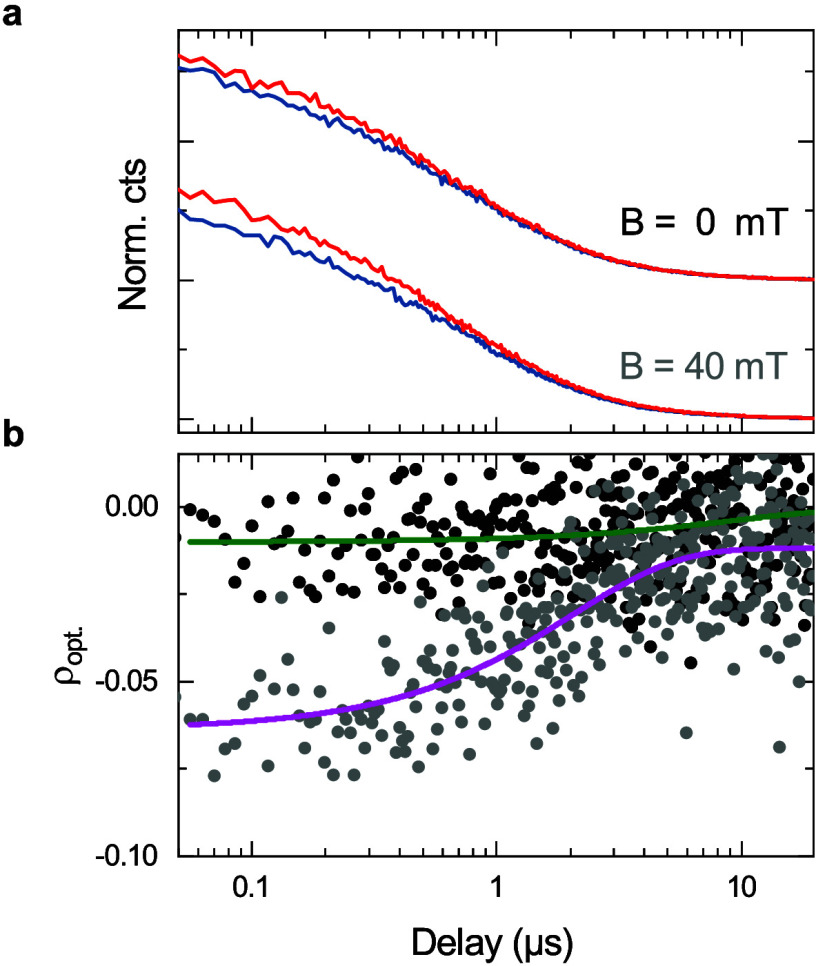
**Temporal evolution of iX valley polarization.** (a) Polarization-resolved iX PL decay acquired at 0 and 40 mT using σ^+^ polarized excitation; blue (red) curve corresponds to PL intensity co- (cross-) polarized with the excitation laser; the data sets are offset for clarity and normalized to the intensity of copolarized component at zero delay. (b) Time-resolved changes of ρ_opt_ for *B* = 0 mT (black) and *B* = 40 mT (gray). The solid curves are guides to the eye.

In conclusion, we showed that iX in 1L-MoS_2_/1L-MoSe_2_ is formed by electrons and holes residing at the edges of the Brillouin zone, with a negligible degree of interlayer hybridization. We find that iX retains its optically induced valley polarization, with the cross-polarized iX PL stemming from the moiré-induced reversal of selection rules. Magnetic field enhances valley-polarization retention by suppressing fast intervalley scattering. The typical magnetic field required for this (≤200 mT) is within reach of a variety of readily accessible techniques, including assembling heterostructures on magnetic substrates^60^ or using rare-earth magnets.^61^ In some devices, we observed |ρ_opt_| up to 7% at zero field, allowing for magnet-free operation. The combination of microsecond-long iX PL lifetime and the retention of valley polarization offers the prospect of combining excitonic and valleytronic functionalities in a single optoelectronic device.

## Methods

### Sample Fabrication

All flakes used for the fabrication of the electrically tunable 1L-MoS_2_/1L-MoSe_2_ are produced by micromechanical cleavage of bulk crystals. Bulk TMD crystals are prepared by a flux zone growth method,^62^ and bulk hBN crystals are grown by the temperature-gradient method.^63^ Graphite crystals are sourced from NGS. The thickness of exfoliated crystals is estimated using optical contrast^64^ and confirmed using PL and Raman spectroscopy for TMDs under 532 nm (2.33 eV) laser illumination (LabRAM HR Evolution, Horiba) and atomic force microscopy for hBN (Dimension Icon, Bruker). Electrically tunable heterobilayer devices are assembled by deterministic dry mechanical transfer using polymer stamps.^34,35^ θ is identified using polarization-resolved SHG^65^ measured at RT using a custom-built optical setup. The SHG laser is a Chameleon Compact Optical Parametric Oscillator providing ∼200 fs pulses with a repetition rate of 80 MHz centered at 1320 nm. To minimize chromatic aberrations, a linearly polarized laser beam with ∼5 mW power is focused onto the sample using a 40x reflective objective (numerical aperture of 0.5, LMM40X-P01, Thorlabs). Polarization orientation is controlled using a superachromatic half-wave plate (SAHWP05M-1700, Thorlabs) mounted in a motorized rotational mount. Electrical contacts to TMD layers and transparent FLG gates are created by direct laser lithography (LW-405B+, Microtech) with a positive resist (AZ5214E, MicroChemicals) followed by electron beam evaporation (PVD200Pro, Kurt J. Lesker) of 5 nm of Cr followed by 45 nm of Au. The resist excess metal layer is then lifted off by immersion in acetone and isopropanol for 30 min.

### Photoluminescence Measurements

RT PL measurements are performed using a LabRAM HR Evolution Raman microscope under 532 nm (2.33 eV) laser illumination. Helicity-resolved magneto-optical measurements are done in a close-cycle bath cryostat (Attodry 1000, Attocube) equipped with a superconducting magnet at a nominal sample temperature of 4 K. Excitation and collection light pass through a home-built confocal microscope in reflection geometry, with a 0.81 numerical aperture apochromatic objective (LT-307 APO/NIR/0.81, Attocube). The PL measurements are taken using 638 nm (1.94 eV) continuous-wave excitation (MCLS1-638, Thorlabs), with incident power below 5 μW. The PL signal collected in epi-direction is isolated using a long-pass filter (FELH0700, Thorlabs) and detected by a 0.75-m spectrometer (SpectraPro 2750, Princeton Instruments) with 150 l mm^–1^ grating and a nitrogen-cooled CCD camera (Spec-10, Princeton Instruments). Time-resolved measurements are performed using a single-photon avalanche photodiode (SPCM-AQRH-16-FC, Excelitas Technologies) and a time-to-digital converter (quTAU, qutools GmbH) with a 81 ps timing resolution. For these measurements, the intensity of the CW laser is modulated using an acousto-optic modulator (MT350-A0.12-VIS, AA Opto Electronic), producing 200 ns pulses with the 100-kHz repetition rate. A dual-channel source meter (2612B, Keithley) is used for electric field tuning.

## Supporting Information Available

Additional data and analysis, including table with the summary of device parameters, Raman spectra, identification of stacking configuration, room-temperature photoluminescence spectra, second harmonic generation plots, atomic force microscopy characterization, Stark tuning for additional devices, summary of photoluminescence lifetimes, g-factors, and magnetic-field dependence of optically induced valley polarization for the six closely aligned devices used in the study (PDF)
