# Supplementary material for: Nature of Long-Lived Moiré Interlayer Excitons in Electrically Tunable MoS2/MoSe2 Heterobilayers
Source: Nano Lett. 2024 Aug 30;24(36):11232–8. doi: 10.1021/acs.nanolett.4c02635 (PMC11403766; doi:10.1021/acs.nanolett.4c02635)
Supplement: Supplementary file 1 — nl4c02635_si_001.pdf [file nl4c02635_si_001.pdf]

Supplementary Information for Nature of long-lived moiré interlayer excitons in electrically tunable $MoS_2/MoSe_2$ heterobilayers

Evgeny M. Alexeev,[*,†,‡] Carola M. Purser,[‡,†] Carmem M. Gilardoni,[‡] James Kerfoot,[†] Hao Chen,[†] Alisson R. Cadore,[†,¶] Bárbara L.T. Rosa,[†] Matthew S. G. Feuer,[‡] Evans Javary,[‡,§] Patrick Hays,[‖] Kenji Watanabe,[⊥] Takashi Taniguchi,[#] Seth Ariel Tongay,[‖] Dhiren M. Kara,[‡] Mete Atatüre,[*,‡] and Andrea C. Ferrari[*,†]

†*Cambridge Graphene Centre, University of Cambridge, 9 JJ Thomson Avenue, CB3 0FA, Cambridge, UK*

‡*Cavendish Laboratory, University of Cambridge, JJ Thomson Avenue, Cambridge CB3 0HE, UK*

¶*Brazilian Nanotechnology National Laboratory, Brazilian Center for Research in Energy and Materials, Sao Paulo, 13083-100, Brazil*

§*École Normale Supérieure, PSL, 5 Rue D'ulm Paris, 75005, France*

‖*Materials Science and Engineering, School for Engineering of Matter,Transport and Energy, Arizona State University, Tempe, Arizona 85287, United States*

⊥*Research Center for Electronic and Optical Materials, National Institute for Materials Science, 1-1 Namiki, Tsukuba 305-0044, Japan*

#*Research Center for Materials Nanoarchitectonics, National Institute for Materials Science, 1-1 Namiki, Tsukuba 305-0044, Japan*

E-mail: ea529@cam.ac.uk; ma424@cam.ac.uk; acf26@cam.ac.uk

Table 1: **Devices parameters.** Summary of key parameters for the devices investigated in this study: twist angle (θ), fast and slow time constant for iX PL decay (τ_1 and τ_2), effective iX g-factor (g), optically-induced valley polarization in the absence of magnetic field and at saturation (ρ^0_{PL} and $\rho^{\mathrm{sat.}}_{\mathrm{PL}}$), and saturation magnetic field for ρ_{PL} ($B_{\mathrm{sat.}}$).

Device #	θ,°	τ_1, μs	τ_2, μs	g	ρ^0_{PL}	$\rho^{\mathrm{sat.}}_{\mathrm{PL}}$	$B_{\mathrm{sat.}}, T$
1	4	0.03	0.13	+6.0	-0.07	-0.13	3
2	1	1.0	4.4	+6.7	-0.04	-0.11	1.25
3	3	0.14	0.56	+2.5	-0.01	-0.07	0.02
4	1	0.33	2.2	+2.7	-0.03	-0.10	≤ 0.25
5	3	0.3	1.7	+1.0	-0.01	-0.09	≤ 1
6	1	0.8	3.0	+8.0	0.0	-0.06	0.2
7	28	n.a.	n.a.	n.a.	n.a.	n.a.	n.a.
8	27	n.a.	n.a.	n.a.	n.a.	n.a.	n.a.

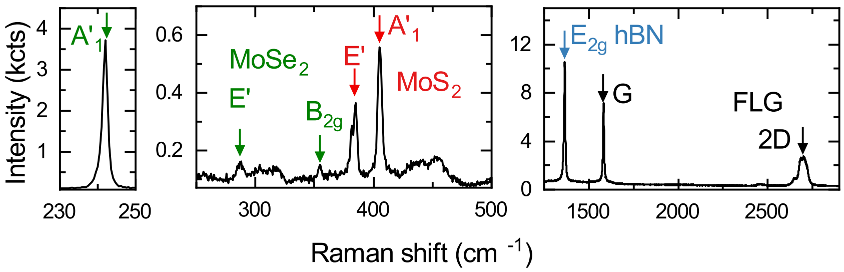

Figure S1: **Raman spectroscopy.** Raman spectra from the device, acquired at room temperature using 2.33 eV optical excitation. Dominant peaks in the 50-500 cm^{-1} range correspond to in-plane A_1' mode at 241.9 (405.4) cm^{-1} and out-of-plane E' mode at 286.7 (384.0) cm^{-1} of $MoSe_2$ (MoS_2).[1] The peak at 354.7 cm^{-1} is out-of-plane B_{2g} mode of $MoSe_2$, which is Raman inactive on monolayers,[2] but becomes active in few-layers and heterobilayers due to the reduction of symmetry elements.[3] Higher-frequency range shows E_{2g} mode at from hBN encapsulation layers at 1365 cm^{-1}[4] and the G and $2D$ Raman modes from FLG gates, at 1582 and ~2697 cm^{-1},[5] respectively.

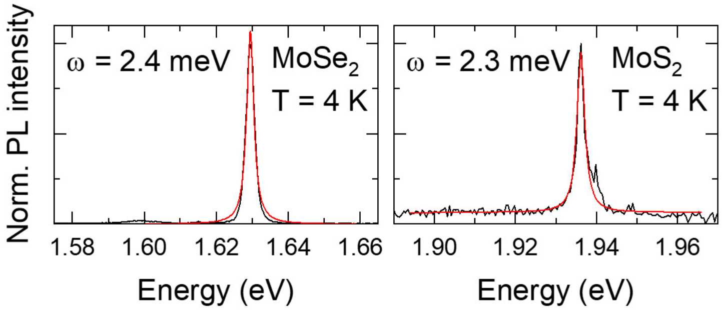

Figure S2: **Photoluminescence spectroscopy of constituent monolayers.** PL spectra recorded in (left) $MoSe_2$ and (right) MoS_2 monolayer regions at 4 K. The neutral exciton peak linewidth approaches the homogeneous limit, confirming the high optical quality.

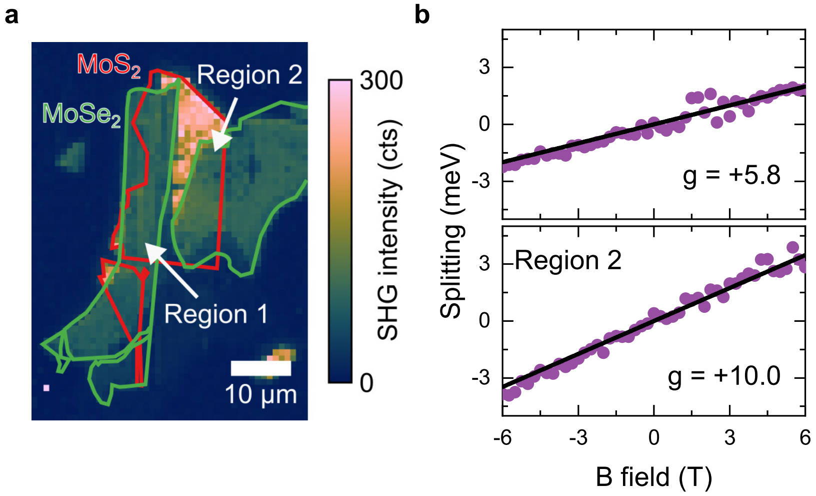

Figure S3: **Stacking configuration identification.** (a) Map of SHG intensity recorded in Device 6 at room temperature using 1320 nm excitation, containing heterobilayer Regions 1 and 2 with the opposite stacking created using tear-and-stack method. Both regions demonstrate similar SHG intensity, preventing reliable identification of stacking configuration. (b) Energy splitting between $\sigma+$ and σ- polarized PL as a function of out-of-plane magnetic field for heterobilayer Regions 1 and 2.

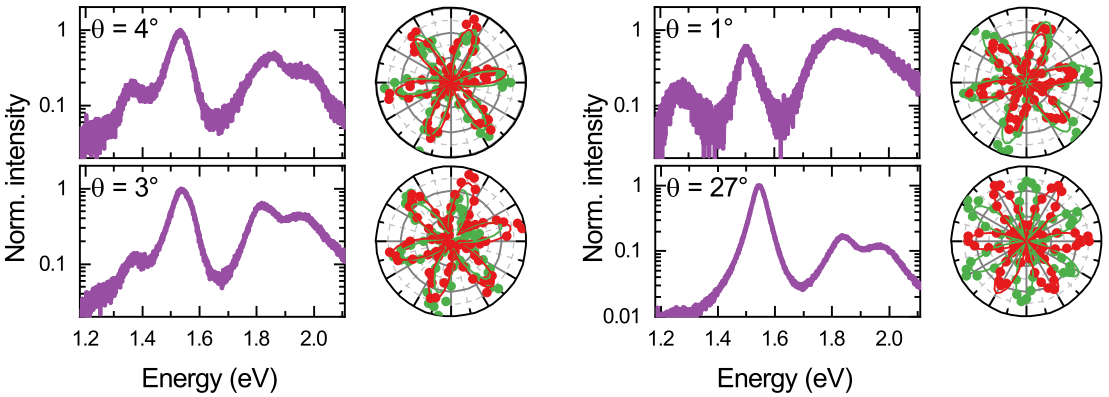

Figure S4: **Twist angle effects.** Normalized PL spectra and polar plots of polarization-resolved SHG signals acquired at room temperature in four devices with different rotational alignment between the TMD layers. Three devices with close rotational alignment ($\theta \leq 5°$) show iX peak in the PL spectrum, which is not present in the PL spectrum of the strongly misaligned device ($\theta \approx 27°$).

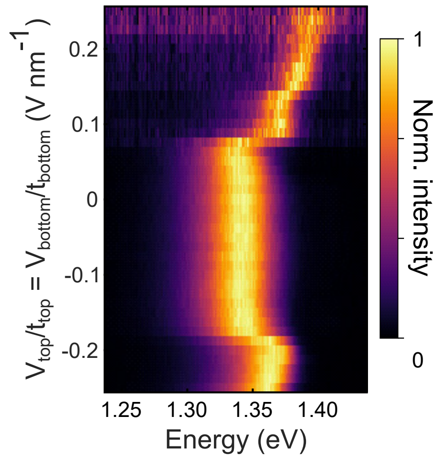

Figure S5: **Doping dependence of iX emission.** Normalized PL spectrum as a function of electrostatic doping of the TMD heterostructure, with $\mathrm{V_{top}}/t_{\mathrm{top}} = \mathrm{V_{bottom}}/t_{\mathrm{bottom}}$. Here, $\mathrm{V_{top}}$ ($\mathrm{V_{bottom}}$) is the gate bias applied between the top (bottom) graphene electrode and the heterostructure, and t_{top} (t_{bottom}) is the thickness of the top (bottom) hBN dielectric.

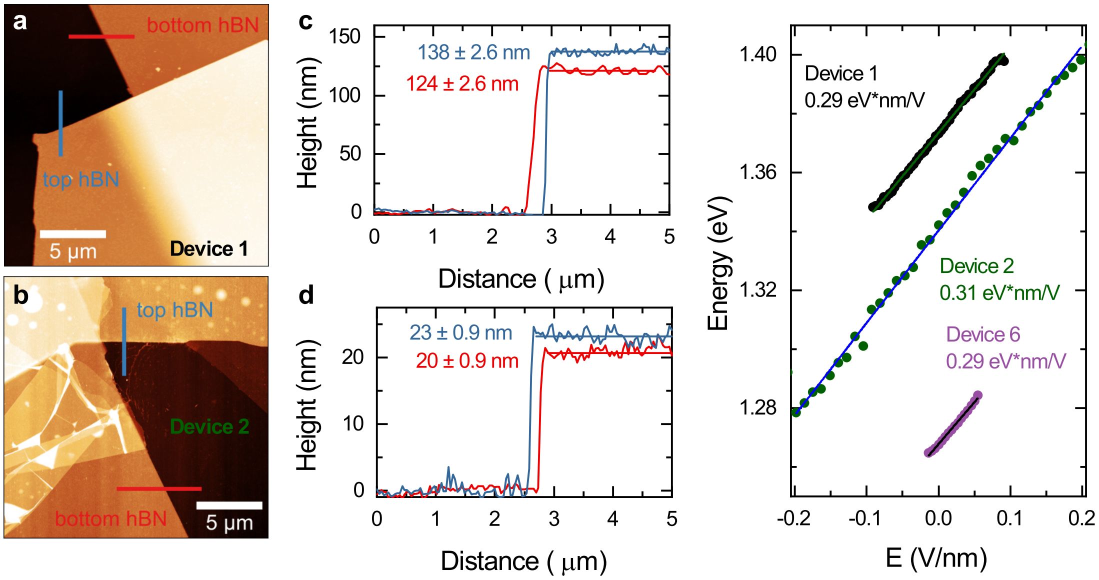

Figure S6: **Dipole size identification.** (a-c) (a and b) AFM topography images of devices 1 and 2 acquired at the edges of top and bottom hBN encapsulation layers and (c and d) cross-sections taken at the positions identified by the corresponding colour lines, showing the thickness of the layers. (e) Change of iX emission energy under applied out-of-plane electric field measured in Devices 1,2, and 6. The rate of change of ~ 0.3 eV V/nm extracted from the linear fit corresponds to the dipole size of ~ 0.55 nm.

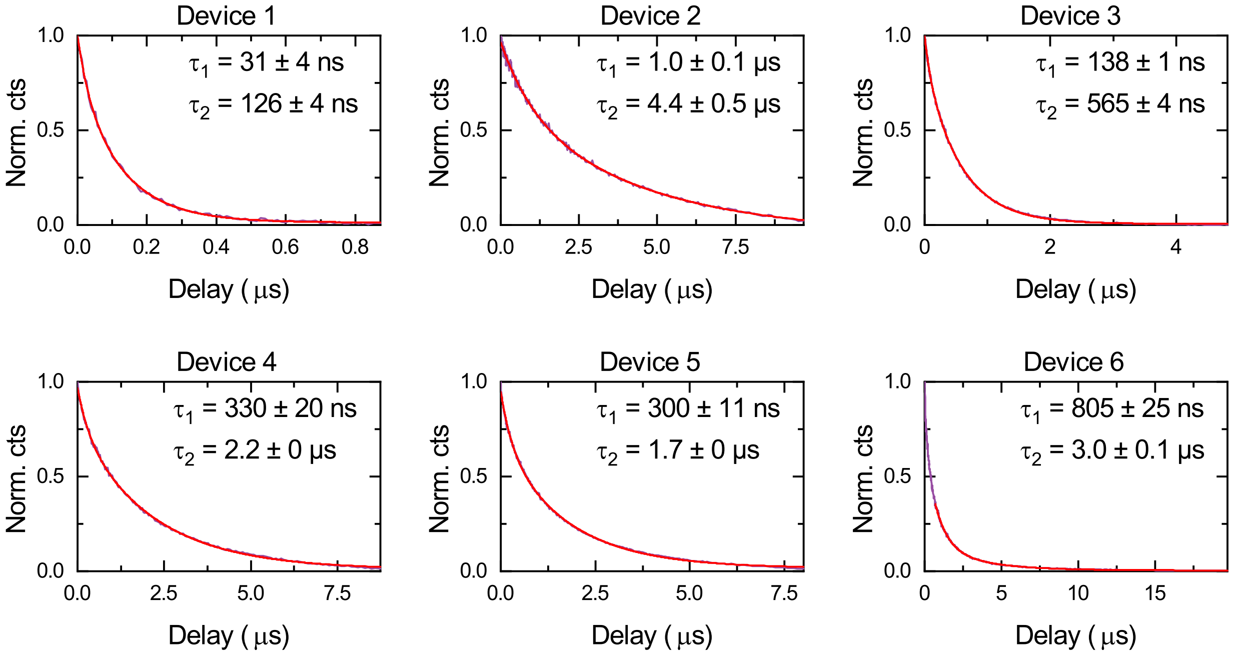

Figure S7: **iX lifetimes.** iX PL decay acquired in Devices 1-6. Solid red lines are bi-exponential fits to the data, time constants extracted from the fit are presented next to each curve.

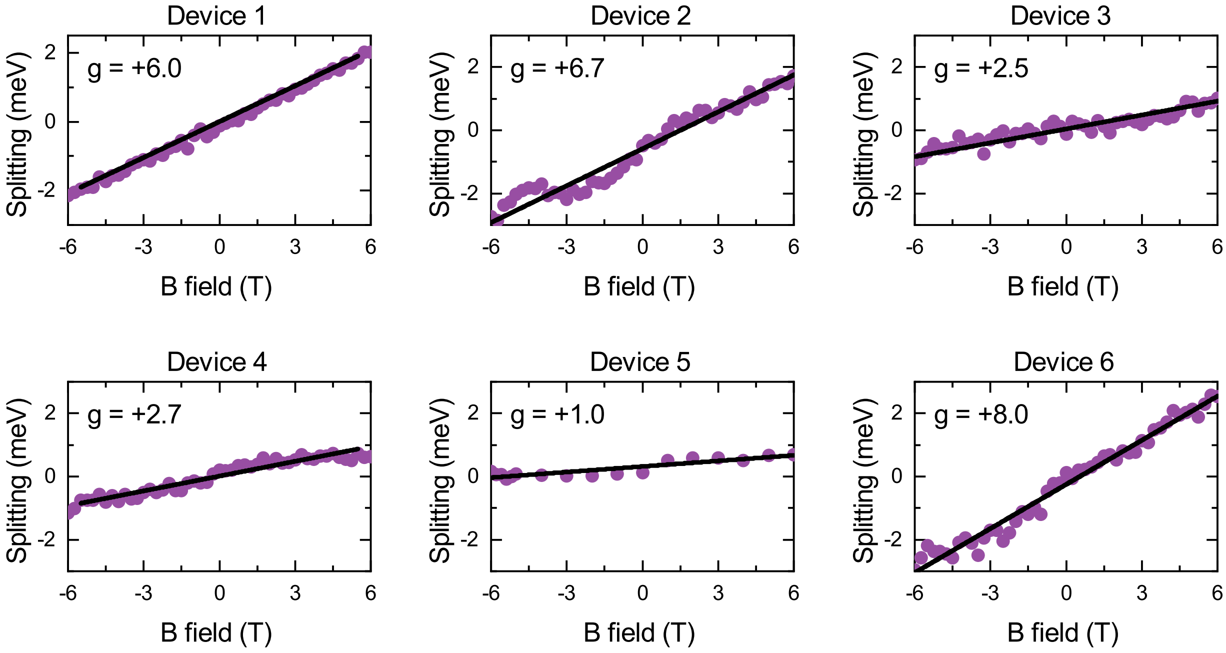

Figure S8: **iX effective g-factors.** Energy splitting between σ^+ and σ^- polarized PL as a function of out-of-plane magnetic field for Devices 1-6. Landé g factors extracted using linear fitting are listed next to each plot.

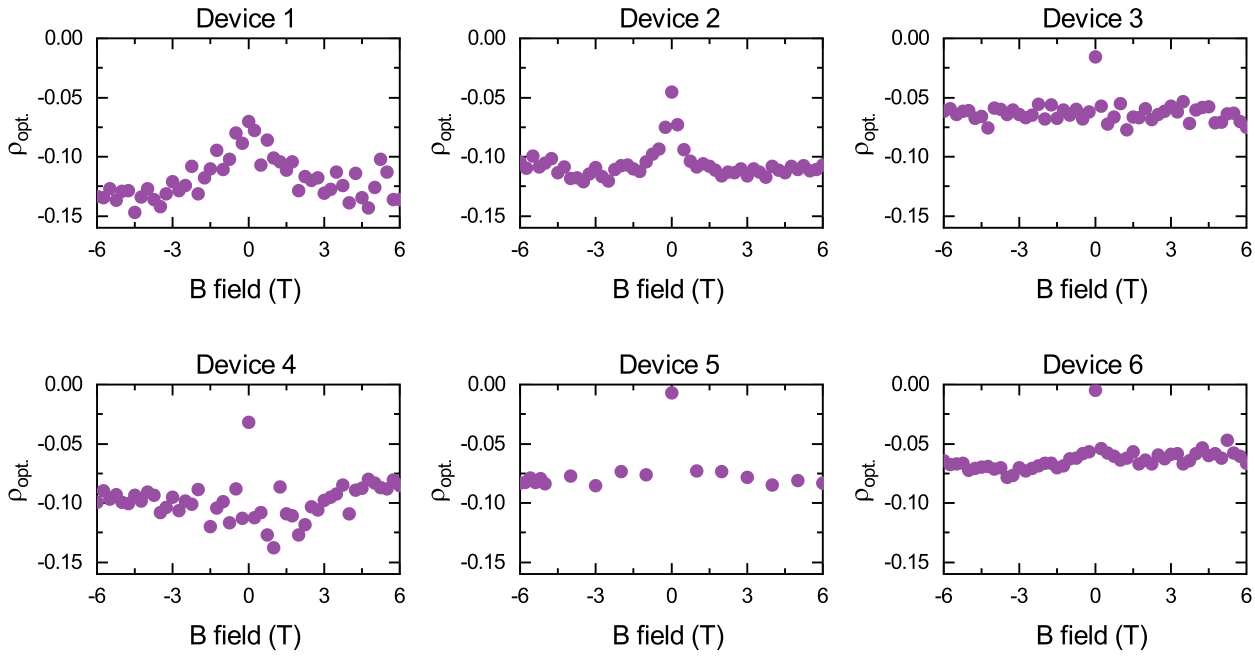

Figure S9: **Optically induced valley polarization.** Changes of optically induced iX PL valley polarization under applied magnetic field for Devices 1-6.

References

(1) Terrones, H.; Del Corro, E.; Feng, S.; Poumirol, J. M.; Rhodes, D.; Smirnov, D.; Pradhan, N. R.; Lin, Z.; Nguyen, M. A.; Elías, A. L.; Mallouk, T. E.; Balicas, L.; Pimenta, M. A.; Terrones, M. New First Order Raman-Active Modes in Few Layered Transition Metal Dichalcogenides. *Sci. Rep.* **2014**, *4*, 1–9.

(2) Tonndorf, P.; Schmidt, R.; Böttger, P.; Zhang, X.; Börner, J.; Liebig, A.; Albrecht, M.; Kloc, C.; Gordan, O.; Zahn, D. R. T.; Michaelis de Vasconcellos, S.; Bratschitsch, R. Photoluminescence Emission and Raman Response of Monolayer MoS2, MoSe2, and WSe2. *Opt. Express* **2013**, *21*, 4908.

(3) Pan, Y.; Li, S.; Rahaman, M.; Milekhin, I.; Zahn, D. R. T. Signature of Lattice Dynamics in Twisted 2D Homo/hetero-Bilayers. *2D Mater.* **2022**, *9*, 045018.

(4) Reich, S.; Ferrari, A. C.; Arenal, R.; Loiseau, A.; Bello, I.; Robertson, J. Resonant Raman Scattering in Cubic and Hexagonal Boron Nitride. *Phys. Rev. B - Condens. Matter Mater. Phys.* **2005**, *71*, 1–12.

(5) Ferrari, A. C.; Meyer, J. C.; Scardaci, V.; Casiraghi, C.; Lazzeri, M.; Mauri, F.; Piscanec, S.; Jiang, D.; Novoselov, K. S.; Roth, S.; Geim, A. K. Raman Spectrum of Graphene and Graphene Layers. *Phys. Rev. Lett.* **2006**, *97*, 1–4.
